# Supplementary material for: Exploring infection prevention and control knowledge and beliefs in the Solomon Islands using Photovoice
Source: PLOS Glob Public Health. 2022 Nov 29;2(11):e0000680. doi: 10.1371/journal.pgph.0000680 (PMC10021726; doi:10.1371/journal.pgph.0000680)
Supplement: Attachment — Submitted filename: PGPH-D-21-00961 Response to Reviewers.docx [file pgph.0000680.s001.docx]

## PGPH-D-21-00961

### Response to Reviewers

| **Item** | **Section** | **Revision/ Comments** |
| --- | --- | --- |
|  | **Reviewer 1** | |
|  | (General) The article presents the results of original research and was, for the most part, clear and easy to read. The participatory action research method undertaken is a novel and interesting one and was described in sufficient detail within the manuscript. The data collection procedure has also produced results that feed into the discussion and conclusions adequately. | Thank you for your positive overall comments |
| 1 | (Major) Literature speaking to this exact issue seems to be lacking in the current, available literature, however, the paper could be further strengthened by literature speaking to similar issues around the influence of cultural and social beliefs on IPC. For instance, the article could integrate literature on barriers and facilitators to healthcare workers' adherence to IPC guidelines: https://www.cochranelibrary.com/cdsr/doi/10.1002/14651858.CD013582/pdf/full. Similarly, how cultural beliefs may influence HAI in other (institutional, geographic etc.) settings such as the "clean care is safer care" belief presented in this article could be reviewed: https://journals.plos.org/plosone/article?id=10.1371/journal.pone.0140509. There are other articles speaking around this topic and a more exhaustive review of the literature, particularly in the "Culturally centred approaches..." (line 103) section, would further strengthen the introduction/background and discussion sections of the article. | Cochrane review findings were incorporated into the discussion section to strengthen the Discussion, however the review does not specifically discuss LMI settings.  Thank you for suggesting the second article, however the focus of my findings paper is to provide insight into the cultural and spiritual reasons why universal IPC practices and guidelines don’t work in a culturally different LMI country. The article you suggested looks at ‘culture change’ in healthcare a high-income society. I therefore could not utilise the points in this article within my paper. |
| 2 | (Minor) The title is a bit too long and too general. I suggest that the first line before the colon be removed and replaced with something speaking to the use of Photovoice and the research being conducted in the Solomon Islands. This would indicate to the reader that the article explores the topic in one specific geographical context and using the particular method of Photovoice more explicitly. | Title shortened to specify geographical location and method. |
| 3 | (Minor) I would suggest that the authors re-read the manuscript to identify other typographical/grammatical errors but a few that I have identified are on:  • Line 106 – “engage” should be “engaging”  • Line 124 – “any all” should just be “all”  • Line 160 – “covert” should be “convert”  • Line 178 – could abbreviate infection prevention and control as done throughout the rest of the article  • Line 532 – should this line read “to NOT attend”?  • Line 538 – this sentence does not read very clearly as there is a full stop in the sentence beginning with “On the contrary…” | The suggestions have been taken into consideration and the manuscript edited for clarity |
| 4 | (Major) The finding reads: “…mountain people will understand about medical model if it is explained to them”. In light of this, is the issue more about rethinking culture in health-related communication or about adapting IPC to be more culturally sensitive? I suggest that this quote is unpacked in greater detail and that the point is made more explicit. | The paragraph is reframed to make the point more explicit. The issue here is around the junior nurses showing a lack of cultural respect for the mountain peoples beliefs, therefore not adapting their communication across cultures. |
| 5 | (Major) It would be useful to include what the authors see as the difference between “traditional spiritual frameworks” and “spiritual systems” by adding more information here. | Words have been changed in this sentence to differentiate between traditional spiritual frameworks (a worldview) and introduced spiritual systems (introduced religions such as Christianity). |
| 6 | (Major) It is not very clear how the potato and rat finding is an example of how “Melanesian culture reappeared as a reason for staff sickness”. If there is a reference to how the potato and rat are an example of Melanesian culture in the literature, I suggest that more information is added in this section. If not, perhaps this section should be reframed to make the finding more clear. | This paragraph has been altered to provide clarity for the reader. |
| 7 | (Minor) It is indicated that other studies have been done in this area in other settings. The references for these studies should be included in this sentence. | An example has been included here with references added. |
| 8 | (Major) It is mentioned that there are "only very few studies investigating how spiritual and cultural beliefs impact HCWs perception of infection transmission and subsequently how these perceptions influence practice." I suggest that these studies' findings are described in more detail here and that references be included. | A brief description has been included along with references. |
| 9 | (Major) It is not very clear what is meant by “physical entity” in this section. What “other spiritual entities” are being referred to here? What is the significance of highlighting this separateness/independence of the germs, faeces etc. in this context? Additionally, which “cultural processes” are being referred to? This section is a little vague; some conceptual clarification would provide greater clarity as to what exactly the point is that is being made in this section. | This paragraph has been changed for better clarity around multiple beliefs of disease transmission. |
| 10 | (Major) Beginning from “For staff…”. It would be useful to include why the "juxtaposition of axiological values" is an important consideration in this setting in the Discussion section. As it currently stands, it is mentioned very briefly but is a critical aspect of the central finding in this study: that it is important to consider cultural, social and religious beliefs for IPC. Further literature on this could be integrated to strengthen this important point. Grant and Cain's article on how healthcare workers construct spiritual meaning to bridge science and religion can be found here https://onlinelibrary.wiley.com/doi/full/10.1111/jssr.12285?casa_token=T3pJOyxcqdcAAAAA:xV4Iv8tLTtfffotGw9s4jPS0CC3_ZeQJsjI-ygSdwWr4d8W6TOc-CHPyqo5pG5iZDgfjVpxi18GfS14U. Other authors have also written about this important debate; tying the findings to this literature will greatly improve the Discussion section and highlight the less obvious findings that have been presented in the Findings section. | This paragraph has been altered to provide a clearer picture of the opposing values between Christianity, the Theological governance and biomedicine. |
| **Reviewer 2** | | |
| **Introduction & Rationale** | | |
| 1 | The introduction comprehensively covers important content areas of relevance to the study and provides a good description of the context. | Thank you for your positive comments in this section. |
| 2 | The conceptual orientation is less well described. Starting P5. Line 125. The binary positioning of ‘traditional/folk medicine’ with ‘modern’ (presumably western biomedicine?) is problematic and oversimplifies medical pluralism. Delete the word ‘both’ and replace ‘modern’ with western biomedicine if this is what is meant here. Suggest some nuancing of the discussion of medical pluralism, with perhaps some more specific examples from the literature, to show how different peoples and contexts negotiate multiple beliefs about health and infection prevention in a hegemonic biomedical (and possibly also evangelical Christian) context. | Changes to wording has been made as suggested. I have made changes to the paragraph to further expand on medical pluralism. |
| 3 | No explicit theoretical orientation is detailed for this study. Given the focus on culture and health, did concepts from critical medical anthropology, for example, inform the study? Other critical theory? If so elaborate. | The following sentences were added in the method section as further explanation.  The participatory methodology was supported by a critical qualitative approach. Critical approaches can challenge assumptions and expectations of normal practice that have built over time. Often, HCWs have the impression that their work conditions, and the social and political injustices they face cannot be changed (18). However critical theories aim to integrate theory and practice so that people become aware of discrepancies in their social practices and are motivated to change them. |
| 4 | The hospital history provided is compelling and very interesting, with staff identified as medical missionaries. There are then two overlapping hegemonic paradigms of health and disease transmission operating in this setting -biomedical (IP&C) and Evangelical Christian. It is not clear how the study grappled with the distinctions and intersections and how they might play out in association with client/patient population beliefs about health and experience accessing services. | This manuscript describes the findings of this study and does not aim to provide an in-depth analytic discussion. A discussion paper would be more appropriate to discuss this comment. |
| 5 | This section should conclude with a clear statement of the research objective and any associated research questions. | This is described in the last paragraph, however the paragraph has been altered to make it clearer. |
|  | **Methods** | |
| 6 | There is limited elaboration of the CBPR context of this study. Who were the partners? What was their role in the study at all stages? Where did the idea for the study come from? What was the role of the lead author/ researcher? Other authors on the paper? | Extra paragraphs have been added to the method section to explain the role of the co-researchers and the additional authors. |
| 7 | Photovoice methods are described here and well rationalized. Later, in the section on data collection, photo elicitation appears. The method should be described and rationalized in the methods sections as well. | This is further described in the methods section. |
| 8 | Two phases of data collection approximately a year apart are discussed but not explained. What was the purpose of the two-phase approach? Why these two time periods? Why photovoice in one phase and photo elicitation in the second? | The following text has been added.  This allowed time between the two phases for interview translation, transcription and data analysis. Participant validation occurred prior to the second round of interviews where participant photos were presented back to the participants, along with interview transcripts to verify the correctness of phase one interviews. |
| 9 | The grouping of participants by role is rationalized. The level of detail about each individual participant in each phase of the study presented in tables is not needed and has enough information that it would be possible to identify individual participants and link some of them with the quotes presented in the results. Instead, consider summarizing participant information more generally, perhaps according to the groupings used in analysis. | Contents of the participant tables were converted to text and the tables deleted. |
|  | **Data Collection** | |
| 10 | Elaboration on the implementation of photovoice is needed. Participant groups were given cameras for 48 hours and asked to take no more than 30 photographs. Was each person in the group given a camera and each asked to take 30 photos? Or was a camera distributed among group members for a period of 48 hours, with a collective set of 30 photos produced? Group members were then brought together. Did they collectively choose the 15 photographs for discussion? Were the captions collectively determined? What logistical or other challenges were navigated? | Clarification was provided in this section. |
| 11 | For the photo elicitation phase, elaborate on how this worked. Even though the participants appear to have been different, were the groupings the same? What is meant by the statement (P12. Lines 274-75) that groups were presented with all of the photographs (excluding their own) and asked to choose 5 for discussion? If this group didn’t take photos, what was considered ‘their own’? What was excluded and why? How many photographs were participants asked to sort through in the allotted time period? | The rationale for Photo-elicitation is explained further in this section |
| 12 | The data collection section identifies that project data include photographs and interview transcripts. A reference to field note data appears in a different section of the paper (analysis section), but is not explained or rationalized. | Sentence added - Field notes of interviewee responses were taken using the above framework to assist with data triangulation |
|  | **Data Analysis** | |
| 13 | Some elaboration is needed. Who participated in the analysis? What was the role of co-authors? Community partners? Which photos were included in the NVIVO analysis? Rationale? Inclusion/exclusion criteria? Given that data was collected by groups, was there within and between group analysis? Why/why not? How were content, context, and conceptual orientation included in analysis or interpretation? Was gender attended to? Why/why not? | Edits have been made throughout the paper which addresses the reviewers concerns here. |
|  | **Results** | |
| 14 | Figure 1 is difficult to read and does not add much more information than is already provided in the description that precedes it in the text. | Figure 1 shows the complexity of the influencing factors as to why IP&C doesn’t work at AAH and is therefore a visual representation of this, however I agree that it is a difficult diagram to follow so have removed it. |
| 15 | Some orientation to the presentation of findings under theme 1 (the focus of this paper) would be helpful. Are the headings in this section sub-themes? Interpretive? | Unsure of this comment as theme 1 is presented under 2 subheadings and this is explained. |
| 16 | Good use of quotes | Thank you |
|  | Some questions about the results: What explanation is provided for inadequate disinfection, such as dirty water, reuse of hand towels, uncleaned toilets (P15-16, Lines 354-360; 366-377; 375-77), and other breaches of hygiene practices? How did participants answer the opportunities questions? | Explanation for these practices can be found in the quotes from participants. Opportunities for improvement are outside the scope of this paper as it only provides results of theme 1 – beliefs around sickness transmission. |
| 17 | P19. Lines 456-457. It is not clear how social relations played out as a reason for staff sickness. Where the social relations appear to come into play is in the obligation to treat sickness. | This paragraph has been changed for clarification. |
| 18 | It is not clear how the quote about toileting practices (P.20, lines 478-482) is explained as a cultural difference. What makes this ‘cultural’ rather than contextual? | This sentence has been edited for clarity. |
|  | **Discussion** | |
| 19 | Ensure no new uncited findings are brought up in the discussion E.g. P22. Lines 527-530. | The discussion highlighted here as being uncited – Results can be found on page 19, line 436. This was a finding of the study, however a photo has not been supplied. |
| 20 | Given this is PAR, some discussion of the action implications at a practical level would strengthen the manuscript. What opportunities do participants identify for biomedical issues identified? For cultural/spiritual issues? Is there support for or resistance to an IP&C strategy that attempts to accommodate the health and disease beliefs revealed in this study? What role does the ‘medical missionary’ context play in possible opportunities? | This paper describes the findings of one component of the study, and a sentence has been added to the end of the data analysis section which now highlights this. The reviewer’s questions are valuable however are better answered in future papers from this study. |
| 21 | It would be interesting to discuss some of the overlaps that may have been revealed, for example, rubbish handling may have implications for the biomedical and cultural/spiritual perspectives on disease transmission. | Edits have been made throughout the manuscript which addresses the reviewer’s concerns |
| 22 | Elaborate individual and collective consent, especially with reference to the photographs. In what ways did participants and the community-based partner agree to the use of the photographs? For data analysis purposes? For use in academic or public presentations or publications? Was there a photo release process? Example: The person in figure 3 is potentially identifiable. The pixelated face suggests that this person may not have consented to be identified. Clarify photo release circumstances. | Consent for use of photos has been further explained under Consent to participate heading. Participants were instructed not to take photos of people where the person could be identified, as there a ‘consent’ to be photographed was not included in this study. |
|  | **Other methodological and interpretive considerations** | |
| 23 | Strengths, challenges, and limitations are not identified or discussed. | Limitations have been added add the end of the Discussion section. Strengths of the research method has been discussed throughout the method section and challenges have also been interspersed throughout the paper. |
| 24 | No qualitative rigour criteria are identified or discussed with reference to this study. | Statements around research rigour have been added throughout.  Using Photo-elicitation during the second phase enhanced the credibility and trustworthiness of the data as the analysis of the same photograph by different participants assisted with data triangulation.  Field notes of interviewee responses were taken using the above framework to assist with data triangulation with a translator present, and interviews about the photographs were recorded using a digital audio recorder.  Further to this, they live with their reality, therefore input and expertise from the hospital staff was essential in the study design, and this also moderated the primary researcher’s reflexivity.  Participant validation occurred prior to the second round of interviews where participant photos were presented back to the participants, along with interview transcripts to verify the correctness of phase 1 interviews. |
| 25 | Edit suggestions.  P2. Line 46. Cite SENIC  P5. Lines 105-107. Cite examples of this statement. | SENIC has been cited  P5 Lines 105-107 have been further explained throughout this section. |
| 26 | The paper could benefit from an edit review.  Some of the errors noted:  P4-5. Lines 99-102. Beginning “Not one that is found…” Incomplete sentence  P5. Line 112. Insert comma after ‘transmission’  P5. Lines 113-115. Awkward sentence.  P5. Line 124. Delete ‘any’  P5-6. Lines 125-127. Beginning “Medical pluralism…” Incomplete sentence  P7. Line 177. Following ‘members’ delete ‘to’  P15. Line 336. Change ‘was’ to ‘were’.  P23. Lines 538-539. Beginning “On the contrary…” Incomplete sentence  P23. Lines 559-560. Beginning, “Nor can researchers...” Incomplete sentence | Revisions have been made as suggested. |
|  | **Reviewer 3** | |
| 1 | Thanks to the authors for choosing an interesting method regarding Infection Prevention. However, I think the abstract does not reflect the results well. hence, this should be rewritten. | Thank you for the complement. The abstract has been altered. |
| 2 | In the **method** section, the study site and study design should be explained with more clarity. Table 3 is mentioned in the data analysis section but is presented in the result section. This should be corrected. | The method section has been enhanced. The study site is described in the Research Context section. Table 3 and the associated explanation has been moved into the data analysis section. |
| 3 | The result section is comparatively well written, however, each domain or theme needs to be supported by facts, which was not found in all the cases. | Could I please seek clarity on this recommendation? The paper explains that results from theme 1 only are presented. |
| 4 | The findings in the discussion, in some occasions, were not compared with the literature available. The authors are advised to have a recheck on this. | The Discussion section has been revised with additional discussion and references added. |
